# Supplementary material for: Predicting visual function by interpreting a neuronal wiring diagram
Source: Nature. 2024 Oct 2;634(8032):113–23. doi: 10.1038/s41586-024-07953-5 (PMC11446822; doi:10.1038/s41586-024-07953-5)
Supplement: Supplementary file 6 — CRF and ERF predictions for individual TmY4 and TmY9 cells. Analogous to Supplementary Data 3, but for TmY target types. Shown are the top four monosynaptic pathways, the strongest pathway passing through each of the top ten intermediary types (ranking from Extended Data Fig. 7), and the trisynaptic pathway Tm1–TmY–Dm3–TmY (see the section entitled Prediction of spatial normalization). [file 41586_2024_7953_MOESM6_ESM.zip › DataS4/TmY9qΓèÑ/720575940626947227.pdf]

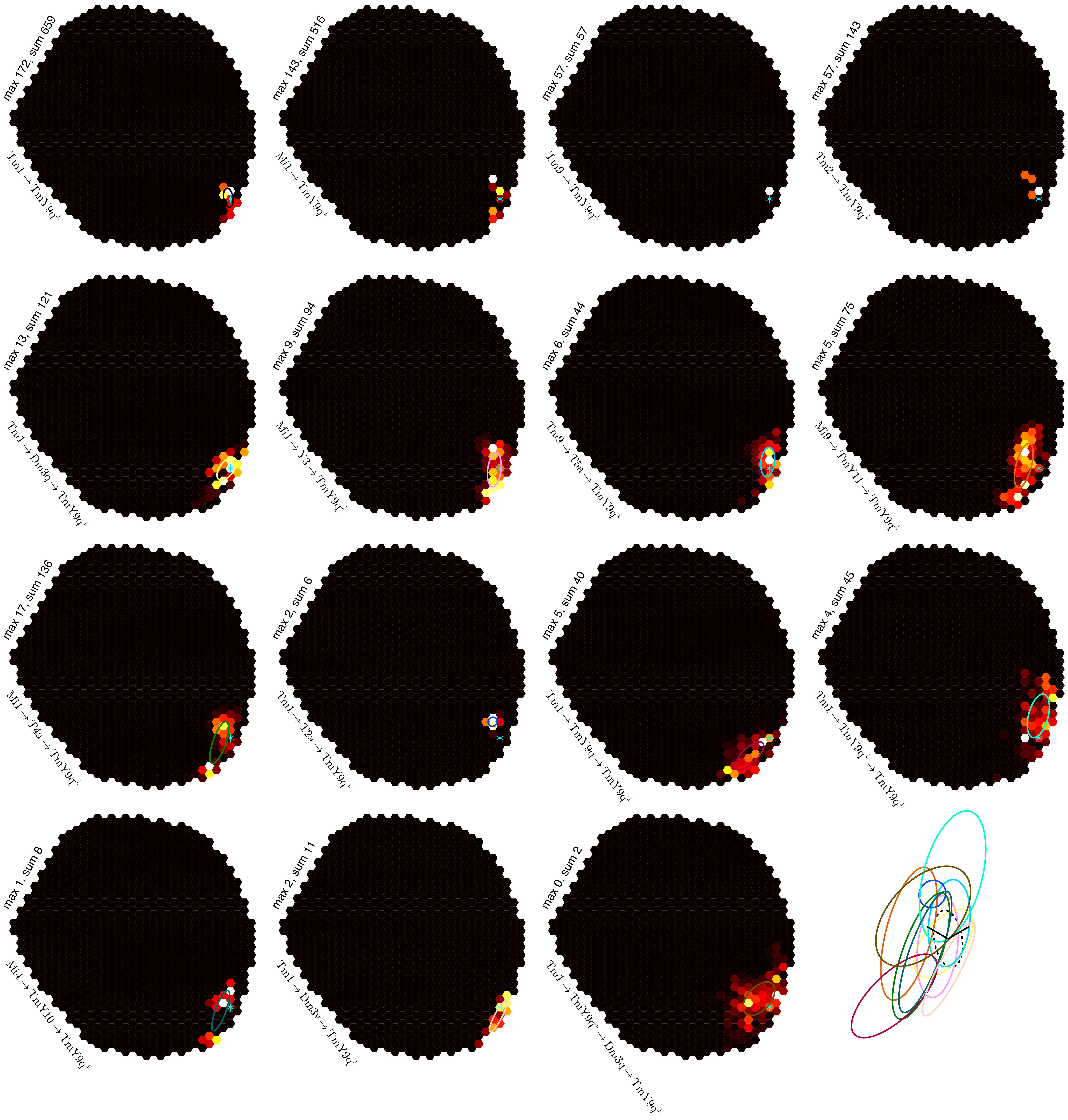

max 172, sum 659
Tm1 → TmY9q⊥
max 143, sum 516
Mi1 → TmY9q⊥
max 57, sum 57
Tm9 → TmY9q⊥
max 57, sum 143
Tm2 → TmY9q⊥
max 13, sum 121
Tm1 → Dm3q → TmY9q⊥
max 9, sum 94
Mi1 → Y3 → TmY9q⊥
max 6, sum 44
Tm9 → T5a → TmY9q⊥
max 5, sum 75
Mi9 → TmY11 → TmY9q⊥
max 17, sum 136
Mi1 → T4a → TmY9q⊥
max 2, sum 6
Tm1 → T2a → TmY9q⊥
max 5, sum 40
Tm1 → TmY9q → TmY9q⊥
max 4, sum 45
Tm1 → TmY9q⊥ → TmY9q⊥
max 1, sum 8
Mi4 → TmY10 → TmY9q⊥
max 2, sum 11
Tm1 → Dm3v → TmY9q⊥
max 0, sum 2
Tm1 → TmY9q⊥ → Dm3q → TmY9q⊥
